# Infant mortality rate and time to death determinants in East Africa: A Bayesian spatial frailty analysis of DHS data (2015–2022)

**DOI:** 10.1371/journal.pone.0357712

**Published:** 2026-10-05

**Authors:** Tigist Kifle Tsegaw, Rediet Eristu, Dessie Abebaw Angaw

**Affiliations:** Department of Epidemiology and Biostatistics, Institute of Public Health, College of Medicine and Health Sciences, University of Gondar, Gondar, Ethiopia; Kwame Nkrumah University of Science and Technology College of Health Sciences, GHANA

## Abstract

**Background:**

Infant mortality refers to the death of an infant before their first birthday. In 2021, approximately 3.8 million infants died worldwide. While interventions have reduced infant mortality rate (IMR) globally, Sub-Saharan Africa (SSA), particularly East Africa, still faces high IMR. Despite many studies, evidence regarding on the impact of spatial effects remain limited. This study aimed to incorporate spatial random effects to identify factors associated with infant mortality.

**Methods:**

Secondary data analysis was conducted using a total weighted sample of 101,532 infants from DHS data collected between 2015 and 2022 in East Africa. STATA version 14 was used for data cleaning, and R version 4.3.1 was used for data analysis. A Bayesian spatial frailty analysis model was fitted, and convergence was checked using trace plot. The model goodness of fit was assessed using Cox-snell residual plot.

**Results:**

The IMR was 39.83 per 1000 live births (95% CI: 35.81–44.27). Breastfeeding initiation time after 24 hours (HR = 4.033, 95% CrI: 3.869–4.207), not having antenatal care (ANC) follow-up (HR = 1.534, 95% CrI: 1.259–1.869), maternal age between 15 and 24 years (HR = 1.256, 95% CrI: 1.11–1.411), low birth weight (HR = 1.575, 95% CrI: 1.388–1.770), plurality (HR = 4.0, 95% CrI: 3.334–4.746), parity more than ten (HR = 2.173, 95% CrI–1.4413.125), parity between five and ten (HR = 1.264, 95% CrI: 1.075–1.472), being a male child (HR = 1.276, 95% CrI: 1.149–1.411), and maternal employment status (HR = 0.740, 95% CrI: 0.606–0.909) were factors associated with infant mortality. High frailty was detected in northern and southwestern Malawi and in the western regions of Mozambique, Zambia, and Burundi.

**Conclusion:**

The pooled IMR was higher than the global estimate of IMR. Infant mortality was associated with maternal, infant, and reproductive factors. In addition, high spatial frailty was observed in some areas, suggesting the presence of unmeasured regional factors related to geographic location. These findings call attention to the need for policies that focus on strengthening antenatal care coverage, promoting early initiation of breastfeeding, improving maternal socioeconomic empowerment, and prioritizing high-frailty areas through resource allocation to reduce infant mortality.

## 1. Background

Infant mortality refers to the death of an infant before his or her first birthday. The infant mortality rate (IMR) is the number of infant deaths per 1,000 live births and is a key indicator of maternal and child health, health care access, and broader social and economic conditions [1,2].

Globally, infant deaths have declined from 8.7 million in 1990 to 3.8 million in 2021 [3], of which approximately 1.9 million were in Africa [4]. Over the same period, the global IMR has decreased from 65 deaths per 1000 live births to 28 deaths per 1000 live births [4,5]. In Sub-Saharan Africa, it declined from 107 deaths per 1000 live births in 1990 to 46.5 deaths per 1000 live births in 2022 [5]. However, this progress has been slower than that of other United Nations Sustainable Development Goal regions, including middle-income countries [6].

In East Africa, the pooled infant mortality rate was 41.41 per 1000 live births in 2018 [4]. Plurality, low birth weight [7], mode of delivery [8], teenage pregnancy, short birth interval, higher birth order [9], home delivery, unimproved water source, female sex, initiation time of breastfeeding [10], being an uneducated mother, and distance from a health facility were identified as significant factors for infant survival time and infant mortality rate [11].

By 2030, the under-five mortality rate is expected to decline to 25 per 1,000 live births, according to Sustainable Development Goal (SDG) 3 target 3.2 [12]. However, the United Nations Inter-agency Group for Child Mortality Estimation (UN-IGME) 2023 Child-Mortality Report states that Sub-Saharan Africa had a mortality rate for under-five children of 71 deaths per 1,000 live births, with an annual decline rate of 2.9% [13]. The mortality rate is estimated to decrease to 54.53 deaths per 1,000 live births if it continues at this rate, which is above the goal [4]. Enhancing healthcare access, quality, and usage of skilled care has been prioritized to reduce the burden of high infant death rates and achieve the SDG target 3.2 [13].

Despite interventions having reduced infant mortality rates globally [14–16], Sub-Saharan Africa, especially East Africa, continues to have high infant and under-five mortality rates due to factors such as limited access to healthcare services and the widespread prevalence of infectious diseases [6]. In 2023, except for Kenya, all East African countries’ IMRs exceeded the global estimates, with intercountry variation ranging from 85.06 deaths per 1,000 live births in Somalia to 10.4 deaths per 1,000 live births in Seychelles [14].

Although several studies have examined infant mortality and its associated factors in Sub-Saharan Africa and East Africa using shared frailty or spatial models based on Demographic and Health Survey (DHS) data, they interpret clustering effects as random effects without explicitly modeling spatial dependency across neighboring regions. Shared frailty models account for unobserved heterogeneity but do not capture the spatial autocorrelation arising from geographic proximity. In reality, spatial location plays a critical role in infant mortality, acting as a proxy for unmeasured contextual and regional factors, such as regional socioeconomic conditions, healthcare accessibility, environmental exposures, and infrastructure. Therefore, this study extends the existing literature by jointly modeling infant time-to-death and spatial dependency using a Bayesian spatial frailty survival model, allowing the separation of individual-level risk factors from spatially structured contextual effects. By doing so, this study simultaneously identifies unobserved regional hotspots and individual-level risk factors, a distinction that is not captured by shared frailty or random-effect models.

## 2. Methods

### 2.1. Study area and period

The study was conducted in nine East African countries from 2015 to 2022 (Fig 1). The UN sub-region of East Africa consists of 20 countries in the eastern part of Africa. The region faces some of the most significant health challenges globally, with East Africa experiencing a severe needs-based shortage of healthcare workers and wide variations in workforce density among countries [17].

**Fig 1:**
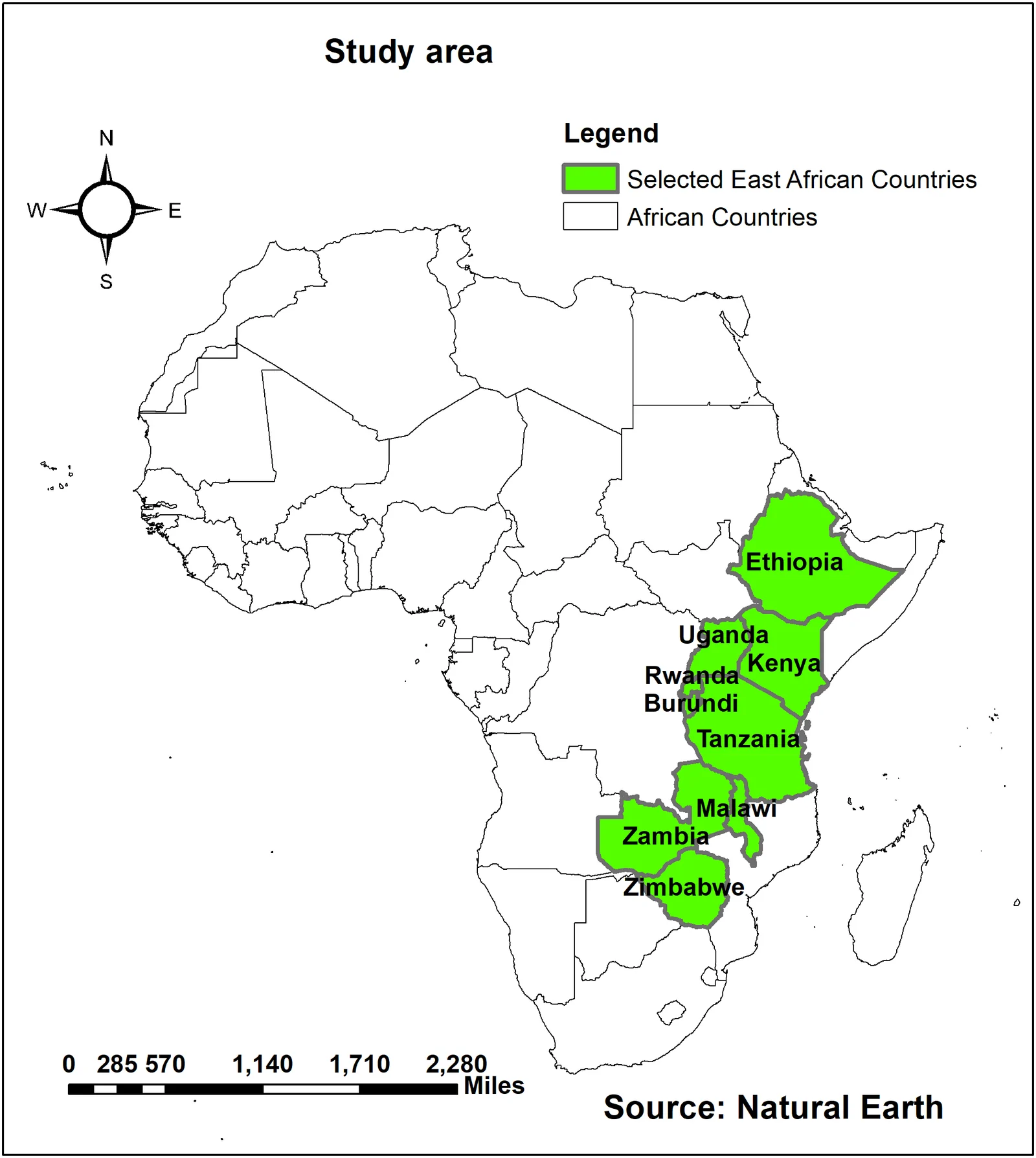
Study area. **Map created using Natural Earth data (public domain):** http://www.naturalearthdata.com**, figure shared under CC-BY 4.0.**

### 2.2. Data source

The data for this study were obtained from the latest DHS surveys conducted in East African countries between 2015 and 2022. A request for the data was submitted, and approval was granted by the DHS program on April 22, 2024. The dataset was downloaded in STATA format from the DHS website (http://www.dhsprogram.com). Countries were selected based on the availability of recent standard DHS data. For this study, we used the Kids Record (KR) files [18].

### 2.3. Population and sampling procedure

All live births born to reproductive-age women within five years preceding the survey in East Africa were the source population. All live births born to reproductive-age women within five years preceding the survey in selected East African countries from 2015 to 2022 were the study population. The DHS program uses a two-stage stratified probability sampling designs. Samples were stratified by geographic region and by urban/rural areas within each region. In the first stage, the primary sampling unit, clusters are selected from the enumeration area. The second stage is a complete listing and selection of a total of 25–30 households from each cluster by equal probability systematic sampling; then the data is collected from each selected household [19]. A weighted sample of 101,532 infants was used for descriptive analysis, while 67,304 last-born children were included in the regression analysis, as many of the health service-related characteristics were collected for the last-born child. DHS sampling weights were incorporated into the model to account for unequal probabilities of selection and ensure population-representative estimates. Spatial clustering was addressed by including an intrinsic conditional autoregressive (ICAR) spatial frailty term, which captures spatial dependence (Fig 2).

**Fig 2:**
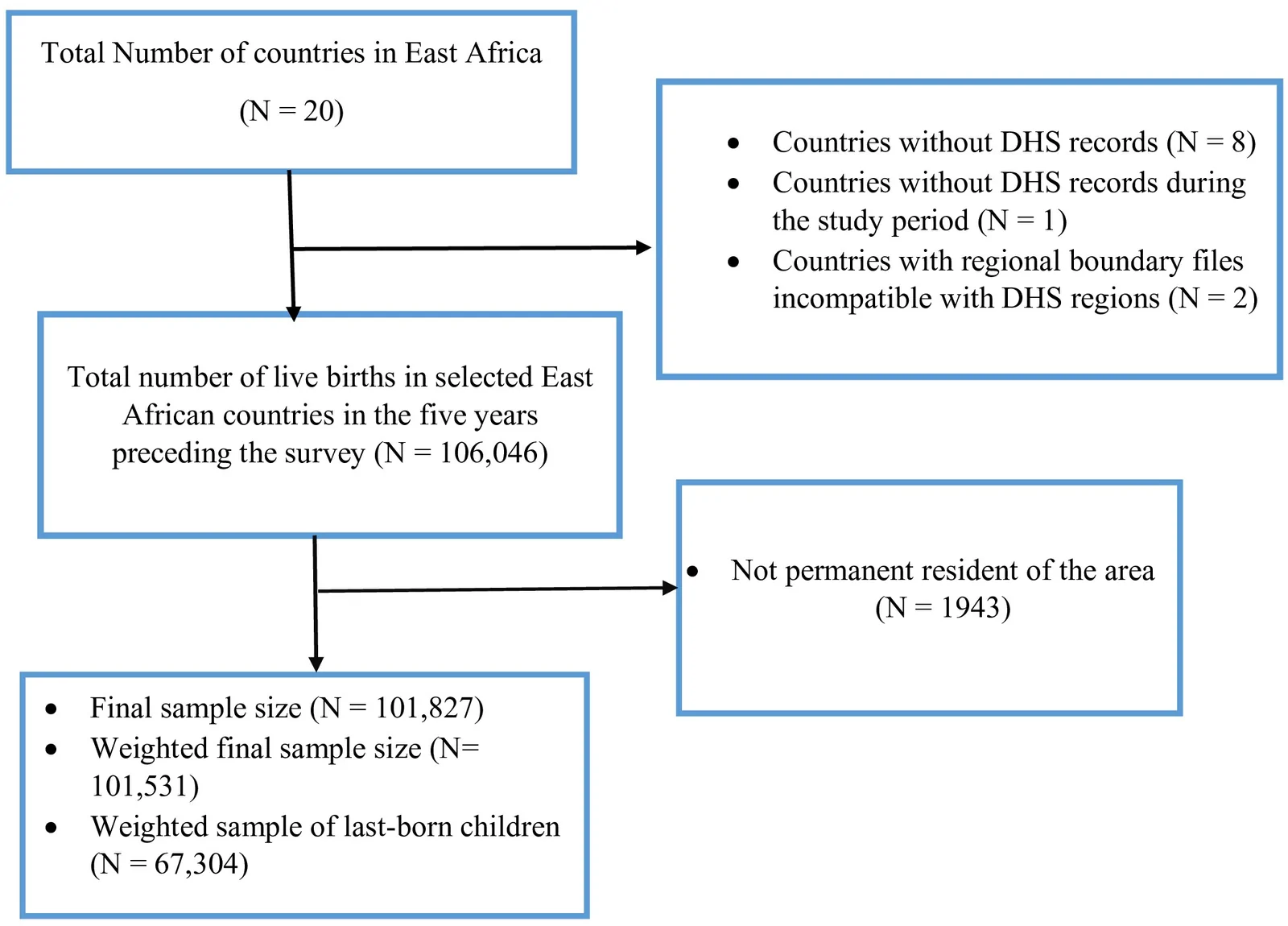
Schematic flow diagram of study participant selection, DHS (2015-2022).

### 2.4. Variables of the study

The outcome variable was time to infant death, measured in completed months from birth up to 12 months of age. In the DHS dataset, age at death is recorded in months, representing the exact month of death rather than a continuous survival time. Therefore, survival time was treated as a discrete-time variable ranging from 1 to 12 months. Infants who died before reaching 12 months of age were coded as having experienced the event (event = 1), with the month of death recorded as the exact failure time. Infants who survived beyond 12 months or who were alive at the time of the survey but had not yet reached 12 months of age were treated as right-censored (event = 0), with censoring occurring at the infant’s age in completed months at the time of interview. Maternal age, marital status, place of residence**,** father education, wealth status, maternal employment, media exposure, antenatal care, birth interval, place of delivery, mode of delivery, parity, birth order, sex, birth weight, time of breastfeeding initiation, distance from health facility, and water supply source were the independent variables.

#### 2.4.1. Operational definition.

**Media exposure:** A mother who reported exposure to television, radio, or newspapers at least once per week was classified as having media exposure [18].

**Distance to health facility**: Women who reported problems in accessing health care for themselves when they are sick are considered to have a problem; otherwise, they don't have a problem [18].

### 2.5. Data processing and analysis

The data were extracted, cleaned, coded, and appended using STATA version 14, and analyzed using R version 4.3.1 software. Descriptive statistics were presented using frequency tables and text. Missing data were managed according to DHS guideline.

#### 2.5.1. Pooled infant mortality rate.

The pooled IMR was calculated using the DHS Rates R package [20]. The DHS divided participants into three cohorts: Cohort A, Cohort B, and Cohort C. Only Cohort B was fully exposed for one year, whereas Cohort A and Cohort C contributed partially. According to DHS guidelines, the exposure time for cohorts A and C should be adjusted, and this was handled using “***DHS.Rates”*** R package for each country, and the infant mortality rate with its standard deviation was computed. Then, a pooled estimate was obtained using a random-effects model and presented in a forest plot.

#### 2.5.2. Descriptive survival analysis.

The overall survival probability of infants was estimated using a life table. To compare survival probabilities across different categorical variables, both the Kaplan–Meier curve and the log-rank test were employed. The null hypothesis for the log-rank test was that there was no statistically significant difference in survival probability between the categorical variables. If the p-value is below 0.05, the null hypothesis is rejected.

#### 2.5.3. Bayesian spatial frailty analysis.

The proportional hazards assumption was initially assessed using the conventional Cox proportional hazards model with global and covariate-specific Schoenfeld residual tests. Evidence of violation of the proportional hazards assumption was observed. Both semiparametric proportional hazards (PH) and accelerated failure time (AFT) formulations were considered using the spBayesSurv framework [21]. The baseline survival distribution was modeled flexibly using a transformed Bernstein polynomial (TBP) prior. Candidate models were compared using the deviance information criterion (DIC) and Watanabe–Akaike information criterion (WAIC).

Both PH and AFT formulations were considered. For the PH model, the covariate-dependent survival function is expressed as [22]:


$$\begin{array}{c} \;S_{\text{X}}(t)=S_{\text{0}}{\left(t\right)}^{\text{exp}(\text{X}\beta )} \end{array}$$ (1)

The model extends to the spatial frailty model by including a spatial effect.


$$\begin{array}{c} h\left(t|X_{i},\;\gamma_{i}\right)=h_{\text{0}\;}(t\;)\;exp({\beta \text{X}}_{i}+\gamma_{i}\;)\;where,\;\gamma_{i}=\;v_{i\;}+w_{i} \end{array}$$ (2)

The term $\gamma_{i}$ incorporates the effects of both heterogeneity via the non-spatial frailty $\upsilon_{i}$ and spatial dependence through the spatial frailty$\;\omega_{i}$ [23]. Three semiparametric models, including Weibull, log-logistic, and lognormal, were fitted with spatial and non-spatial frailty.

**2.5.3.1. Areal data modeling.** Since this study used areal data, the whole study area D was partitioned into 169 East African regions. For the spatial frailty term $\;\omega_{j}$, an intrinsic conditional autoregressive model was used. This model estimates the probability of infant mortality in a given region based on the average values of neighboring regions to capture spatial dependence, which helps to account for the correlation between one region and its neighbor through the adjacency matrix. $\alpha_{ij}=$1 where region i and j share a nontrivial border (i.e., a connected curve of more than one point) and $\alpha_{ij}$= 0 otherwise, then the 169 by 169 matrix A = ${[\alpha }_{ij}]$ square adjacency matrix for the region D was created [22]. The ICAR prior is defined through the set of all conditional distributions as


$$\begin{array}{c} v_{j}|\left\{v_{i}:i\neq j\right\}\sim N(\frac{\sum_{j=\text{1}}^{\text{1}\text{6}\text{9}}e_{ij}v_{j}}{e_{i+}},\;\frac{\tau^{\text{2}}}{e_{i+}})j=\text{1},\;\text{2},\;\ldots ,\;\text{1}\text{6}\text{9} \end{array}$$ (3)

Where $e_{j+}=\sum_{j=\text{1}}^{\text{1}\text{6}\text{9}}e_{ij}\;$the neighbor value for region j,$\;\tau^{\text{2}}$, the scale parameter that controls the variability, $\sum_{j=\text{1}}^{\text{1}\text{6}\text{9}}e_{ij}v_{j}$ the sample mean values of $e_{i+}$the neighboring areal unit frailties, and $\frac{\tau^{\text{2}}}{e_{i+}}$ the conditional variance. The induced prior on v under CAR is improper; the constraint $\sum_{j=\text{1}}^{\text{1}\text{6}\text{9}}v_{j}=\text{0}\;$is used to make it proper. The adjacency matrix was imposed on the model to introduce the spatial dependency for the non-spatial data.

**2.5.3.2.** **Spatial effect.** The posterior mean ICAR frailties of the spatial effect of a child dying before his or her first birthday were mapped. Areas with higher posterior mean ICAR frailty values correspond to a higher hazard of death. The spatial frailty estimates the hazard after adjusting for the effect of other variables and determines the likelihood of hazard of infant mortality without the covariate effect.

**2.5.3.3. Markov chain Monte Carlo (MCMC).** Markov chain Monte Carlo (MCMC) draws samples from the full posterior distribution and then makes inferences using the sample as a representative of the posterior distribution.

Let D be $a_{ij},\;b_{ij},\;x_{ij}{,\;s}_{i}$ where $a_{ij}$ the lowest observation time, $b_{ij}$ the upper observation time, $x_{ij}$ the p-dimensional covariate, and, *s_i_* the spatial location i = 1,…, 169, j= 1,…, $n_{i}$ Then,

The likelihood function:


$$\begin{array}{c} \;L(\theta ,\;\beta ,v)=\;\prod_{i=\text{1}}^{\text{169}}\prod_{j=\text{1}}^{n_{i}}{\left[s_{xij}\left(a_{ij}\right)-s_{xij}(b_{ij)}\right]}^{I\{a_{ij}<b_{ij\}}}{f_{xij}\left(a_{ij}\right)}^{I\{a_{ij}=b_{ij\}}} \end{array}$$ (4)

Where L denotes the likelihood function; θ,β, and v represents the baseline survival parameter, the regression coefficient, and the spatial random effect, respectively.

In the prior specification, we want our data information to dominate the prior distribution by assuming reasonably non-informative priors for all parameters in this model. A total of 30 parameters corresponding to 11 variables were used for this analysis, and the regression coefficient specified a multivariate normal distribution.


$$\begin{array}{c} \beta \sim N_{\text{3}\text{0}}(\text{0},\text{1}\text{0}\text{0}\text{0}) \end{array}$$ (5)

For the baseline survival, the Transformed Bernstein Polynomial (TBP) prior was used. For a positive integer L = 15, we adopted a vague prior gamma distribution for α and a normal distribution for θ for baseline survival [24].


$$\begin{array}{c} S_{O}(.)|\alpha ,\theta \sim {TBP}_{L}\left(\alpha ,S_{\theta }(.)\right),\;\alpha \sim \Gamma (\text{0}.\text{0}\text{0}\text{1},\;\text{0}.\text{0}\text{0}\text{1})\;,\theta \sim N_{\text{2}}(\text{0},\text{1}\text{0}\text{0}\text{0}) \end{array}$$ (6)

For the spatial frailty, an intrinsic conditional autoregressive prior was specified. For parameters τ that control the variability of the spatial random effects, we assigned the vague proper gamma prior distribution with shape parameter 0.001 and scale parameter 0.001 [25].


$$\begin{array}{c} \;({\upsilon \text{1}\ldots .\upsilon \text{1}\text{6}\text{9})}^{\text{T}}|\tau \sim ICAR\left(\tau^{\text{2}}\right),\tau^{-\text{2}}\sim \Gamma (\text{0}.\text{0}\text{1},\text{0}.\text{0}\text{1}) \end{array}$$ (7)

It is carried out through an empirical Bayes approach [26] coupled with adaptive Metropolis samplers [27]. Then the posterior density from the likelihood and the prior becomes


$$\begin{array}{c} p\left(\theta ,\;\beta ,v,\;\alpha ,\tau^{\text{2}}|D\right)\propto L(\theta ,\;\beta ,v).p\left(w_{J}|\alpha \right).p(\alpha ).p(\theta ).p(\beta ).p\left(v|\tau^{\text{2}}\right).p\left(\tau^{\text{2}}\right) \end{array}$$ (8)

Where p(.) represents the prior density

To apply these criteria in MCMC analysis, we used 5000 burn-in iterations and 10000 saved iterations, skipped every 20 iterations, and displayed results every 500 iterations. The model selected various combinations of variables, and the combination with the highest percentage was chosen and used to refit the model.

**Checking convergence**

A bulk effective sample size was explored to ensure enough independent samples for reliable statistical inferences from the MCMC analysis. A higher ESS indicates a sufficient number of independent samples. To further evaluate, trace plots of the sampled values were plotted. Good sampling shows a stable range of values resembling a horizontal band, with no long upward or downward trends; then we have evidence that the chain has converged. The smoothness of the distribution was assessed by a density plot, and an autocorrelation plot was also explored to see the correlation between the drawn sample to ensure that an independent or uncorrelated sample was taken from the posterior distribution [28].

**Model comparison**

The DIC and WAIC [29] were used for model comparison. Lower DIC and WAIC values indicate a better-fitting mode [24]. The model goodness of fit was assessed by Cox-snell plot.

### 2.6. Ethical consideration

An ethical approval letter was obtained from the institutional review board of the Institute of Public Health, College of Medicine and Health Science, University of Gondar, with reference number IPH/2880/2024. This study is based on the existing survey data collected by the Demographic and Health Surveys (MEASURE DHS) project (www.measuredhs.com). Informed consent was obtained from participants by the DHS Program during data collection. Permission to access and use the dataset was obtained from the DHS Program.

## 3. Results

### 3.1. Socio-demographic characteristics of the participants

A weighted sample of 101,532 children born within the five years preceding the survey was included in the analysis. The study population was predominantly rural (77.35%), with nearly half of the children born to mothers aged 25–34 years (47.63%). Most mothers were married (74.20%) and employed (63.64%). Nearly half of the households belonged to the poor wealth category (44.87%) (Table 1).

**Table 1:** Socio-demographic characteristics of the study participants in nine East African countries, DHS (2015–2022).

Socio-demographic Characteristics	Unweighted frequency	Percentage	Weighted frequency	Percentage
**Sex of child**				
Male	51,416	50.49%	51,253	50.48%
Female	50,411	49.51%	50,279	49.52%
**Residence**				
Urban	25,619	25.16%	22,997	22.65%
Rural	76,208	74.84%	78,535	77.35%
**Mother’s employment status**				
Employed	63,211	62.08%	64,617	63.64%
Unemployed	38,616	37.92%	36,915	36.36%
**Mother’s education status**				
No	25,655	25.19%	24,616	24.24%
Primary	47,587	46.73%	48,744	48.01%
Secondary and above	28,585	28.07%	28,172	27.75%
**Mother's age**				
15-24 25-34	29,094	28.57%	29,106	28.67%
	48,143	47.28%	48,363	47.63%
35-49	24,590	24.15%	24,063	23.5%
**Marital status**				
Unmarried	25,629	25.17%	26,195	25.80%
Married	76,198	74.83%	75,337	74.20%
**Wealth index**				
Poor	46,576	45.74%	45,554	44.87%
Middle	18,857	18.52%	19,379	19.09%
Rich	36,394	35.74%	36,599	36.05%
**Media exposure**				
Yes	59,134	58.07%	59,521	58.62%
No	42,693	41.93%	42,011	41.38%
**Father's education**				
No	18,172	23.85%	16,934	22.48%
Primary	50,119	65.78%	50,475	66.99%
Secondary and above	7907	10.38%	7929	10.53%
**Distance to the health facility**				
No problem	55,659	59.97%	61,504	60.69%
Problem	37,158	40.03%	40,028	39.31%

**Single:** living with a partner, never in union, widowed, divorced

### 3.2. Pooled infant mortality rate across East African countries

The overall pooled infant mortality rate was 39.83 per 1,000 live births (95% CI: 35.8144.27) in nine East African countries from the random effect meta-analysis model (Fig 3).

**Fig 3:**
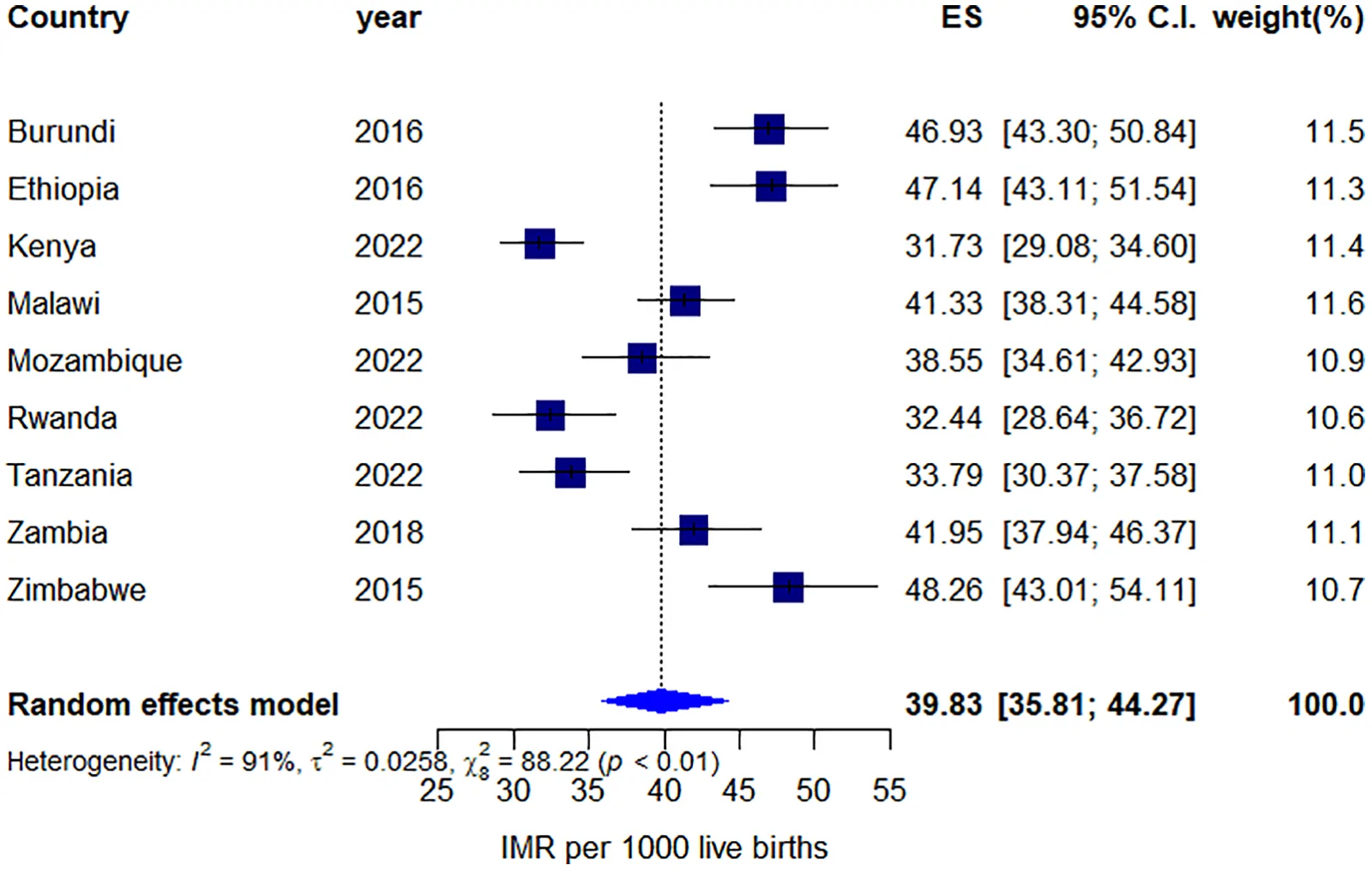
Pooled infant mortality rate in East Africa, DHS (2015–2022).

### 3.3. Bayesian spatial frailty analysis

#### 3.3.1. Nonparametric method.

The inferential analysis included a weighted sample of 67,304 last-born children. The cumulative survival probability declined gradually during the first year of life, with the greatest reduction occurring during the first month after birth. Overall survival decreased from 98.24% at one month to 97.28% at 12 months, corresponding to an overall failure probability of 2.72% (Table 2).

**Table 2:** Life-table cumulative survival probability of infants in East Africa, DHS (2015-2022).

Interval	Number of alive at beginning (Nt)	Number of deaths (dj)	Censored (nj)	Survival probability $(St=\;1-\frac{dj}{Nt})$	overall survival probability [95%CI]
1-2	67,056	1175	3211	0.9824	98.24% [98.10,98.30]
2-3	62,670	78	1689	0.9988	98.08% [97.97,98.18]
3-4	60,903	80	1583	0.9986	97.95% [97.84,98.06]
4-5	59,240	51	1704	0.9991	97.86% [97.75,97.97]
5-6	57,485	36	1713	0.9993	97.80% [97.69,97.91]
6-7	55,736	62	1660	0.9988	97.69% [96.38,97.79]
7-8	54,014	47	1648	0.9981	97.61% [97.48,97.72]
8-9	52,319	51	1723	0.9990	97.51% [97.38,97.63]
9-10	50,545	57	2195	0.9989	97.40% [97.27,97.52]
10-11	48,809	21	1558	0.9993	97.35% [97.23,97.48]
11-12	47,230	36	1775	0.9991	97.28% [97.15,97.40]
>= 12	45442	0	45442	1	97.28% [97.15,97.40]

**Comparison of survival probability**

The Kaplan–Meier curve showed that the overall survival probability of infants decreased and reached around 97.5% at the end of eleven months without accounting for the covariate effects. It dropped sharply in the first month of age and gradually decreased up to 12 months (Fig 4A). Considering gender, the survival probability for both sexes decreased steadily. However, male children had a lower survival probability than female children (Fig 4B)

**Fig 4:**
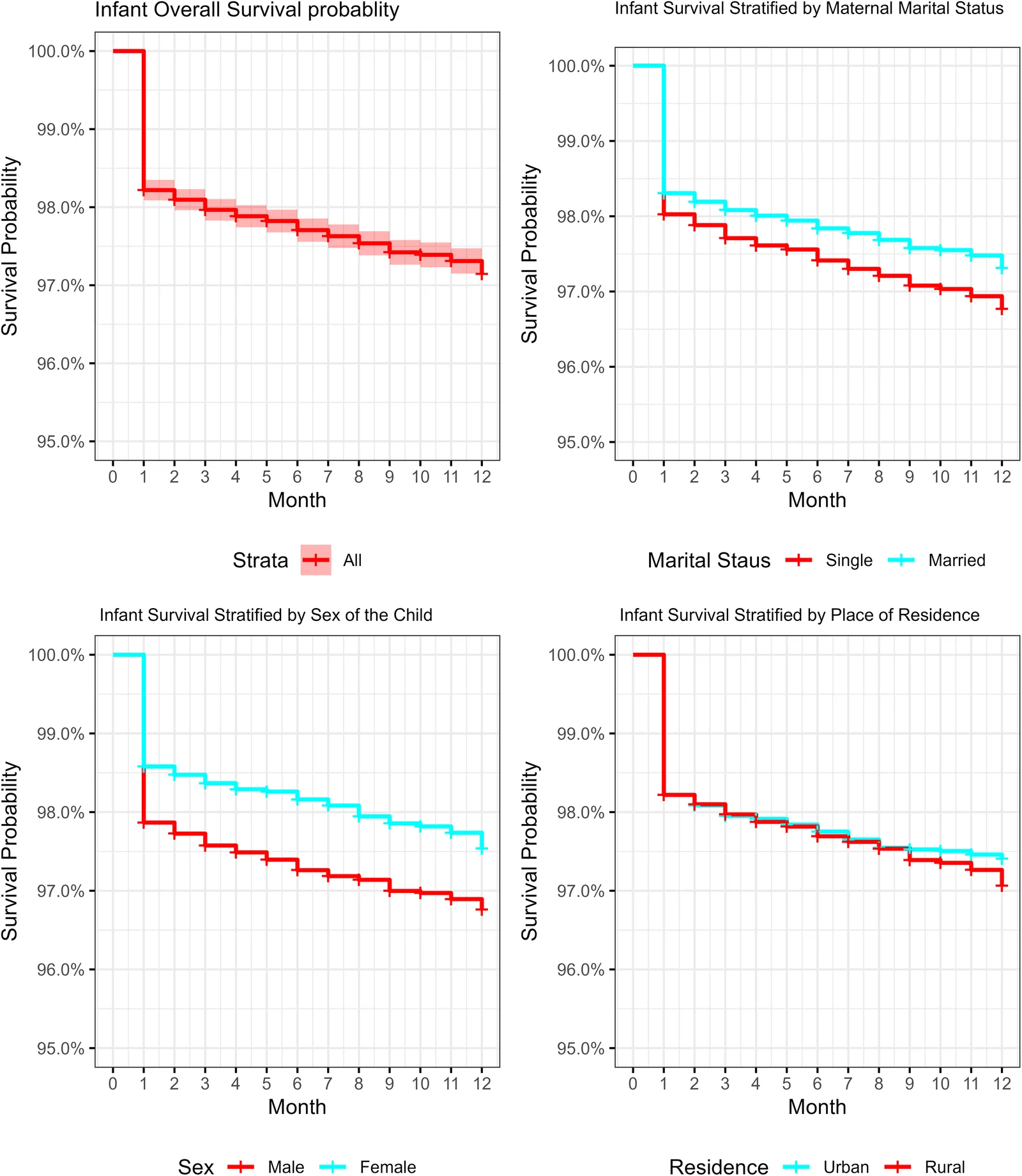
Kaplan–Meier survival estimates for infant mortality. (A) Overall survival probability; (B) Survival by sex of the child; (C) Survival by maternal marital status; (D) Survival by place of residence.

Moreover, from the log-rank test, there was a statistically significant difference in survival probabilities across categories of sex, mother's educational status, age of the mother, marital status of the mother, wealth index of the family, media exposure, number of ANC, parity, place of delivery, plurality, birth interval, breastfeeding initiation time, birth order, mode of delivery, and father's educational status. Conversely, no statistically significant survival probability difference was observed between a place of residence and employment.

**Proportional Hazard (PH) assumption test**

The global Schoenfeld residual test indicated violation of the proportional hazards assumption (p < 0.001). Significant violations were also observed for sex, residence, maternal employment, plurality, distance to a health facility, breastfeeding initiation time, and place of birth, supporting the use of an alternative survival model (S1 Table) [30].

#### 3.3.2. Model comparison.

Seven Bayesian spatial survival models were compared, including lognormal, log-logistic, and Weibull models without spatial frailty, and lognormal, log-logistic, Weibull AFT, and Weibull PH models with spatial frailty. Although the conventional Cox model indicated evidence of non-proportional hazards, both PH and AFT formulations were considered within the Bayesian spatial semiparametric framework. The Weibull PH model with spatial frailty had the lowest DIC and WAIC and was therefore selected as the final model (Table 3).

**Table 3:** Model comparison between spatial and non-spatial frailty with different distributional assumptions.

Model	DIC	WAIC
**Without spatial frailty**		
Lognormal	19,513.63	19,534.78
Log logistic	19,522.76	19,545.5
Weibull	19,508.43	19,525.5
**With spatial frailty**		
Lognormal	19,480.06	19,503.32
Log logistic	19,494.74	19,518.78
Weibull (AFT)	19,463	19,463
**Weibull (PH)**	**18,916.5**	**18,926**

The model selected 17 of 30 parameters, including breastfeeding initiation time, place of delivery, maternal age, size during birth, plurality, sex of the child, place of residence, parity, distance from health facility, and maternal employment status The Cox–Snell residual plot showed departure from the 45° reference line, indicating some discrepancy between the fitted and observed survival distributions this might be due to high censoring of infant as latter months (S1 Fig).

#### 3.3.3. Factors associated with infant mortality rate.

Among the variables included in the final model, eight were significantly associated with infant mortality: breastfeeding initiation time, ANC visits, maternal age, birth weight, plurality, child sex, parity, and maternal employment status. In contrast, place of birth, place of residence, and distance to a health facility were not significantly associated with infant mortality (Table 4).

**Table 4:** Factors associated with infant mortality in East Africa, DHS (2015–2022).

Variables	HR [95%CrI]	ESS
**Breastfeeding initiation time**		
Immediately	Ref	
Within 1 hr.	0.85 (95% CrI: 0.608–1.18)	2866.9
Within 24 hr.	0.922 (95% CrI: 0.787–1.075)	2822.46
After 24 hr.	4.033 (95% CrI: 3.869–4.207)*	1272.4
**Number of ANC**		
No	1.534 (95% CrI: 1.259–1.869)*	2921.1
1-4	1.064 (95% CrI: 0.937–1.201)	2942.87
>5	Ref	
**Mother age**		
15-24	1.256 (95% CrI: 1.11–1.411)*	2984.2
25-34	Ref	
>35	1.135 (95% CrI: 0.983–1.317)	
**Place of birth**		
Home	1.0278 (95% CrI: 0.892–1.181)	2564.6
Health facility	Ref	
**Birth weight**		
Larger	1.098 (95% CrI: 0.971–1.244).	2765.5
Normal	Ref	
Smaller	1.575 (95% CrI: 1.388–1.770)*	2956.3
**Number of pregnancy**		
Single	Ref	
Multiple	4.0 (95% CrI: 3.334–4.746)*	2898.4
**Place of residence**		
Rural	1.035 (95% CrI: 0.916–1.190)	2868
Urban	Ref	
**Parity**		
1-5	Ref	
5-10	1.264 (95% CrI: 1.075–1.472) *	2737.5
>10	2.173 (95% CrI:1.441–3.125) *	2946
**Distance from health facility**		
Problem	1.047 (95% CrI: 0.944–1.135)	2953.86
No problem	Ref	
**Sex of child**		
Male	1.276 (95% CrI:1.149–1.411) *	2576.2
Female	Ref	
**Employment**		
Unemployed	0.740 (95% CrI:0.606–0.909) *	2828.7
Employed	Ref	

Infants who began breastfeeding after 24 hours had 4.033 times a higher hazard of death than those breastfed immediately (HR = 4.033, 95% CrI: 3.869–4.207). Similarly, infants born to mothers with no ANC visits experienced a 53.4% higher hazard of death compared to those whose mothers had more than five ANC visits (HR = 1.534, 95%CrI: 1.259–1.869).

Infants born to mothers aged 15–24 years had a 25.6% higher hazard of death than those born to mothers aged 25–34 years (HR = 1.256, 95% CrI: 1.11–1.411). Likewise, infants with low birth weight experienced a 57.5% higher hazard of death than those with normal birth weight (HR = 1.575, 95% CrI: 1.388–1.770).

Plurality were associated with higher hazard of mortality, with twins, triplets, or quadruplets experiencing 4 times higher hazard of death than singleton births (HR = 4.00, 95% CrI: 3.334–4.746). Male infants also had a 27.6% higher hazard of death than female infants (HR = 1.276, 95% CrI: 1.149–1.411). Furthermore, infants born to mothers with more than ten children had 2.173 times higher hazard of death than those born to mothers with one to five children (HR = 2.173, 95% CrI:1.441–3.125). In contrast, infants born to unemployed mothers experienced a 26% lower hazard of death than those born to employed mothers (HR = 0.740, 95% CrI:0.606, 0.909).

#### 3.3.4. Spatial effect.

The posterior mean ICAR frailty variance was 0.165 (95% CrI: 0.098–0.257), which was statistically significant with a 95% CrI. Fig 5 illustrates the posterior mean of the spatial frailty for infants dying before their first birthday. High frailty values correspond to higher infant mortality. The red color in Fig 5 represents a region with a higher hazard of infant mortality rate, identified in northern and southwestern Malawi, the western region of Mozambique, Zambia, and Burundi. Conversely, the light blue color signifies lower hazard of infant mortality rate, primarily detected in Kenya and central Tanzania (Fig 5).

**Fig 5:**
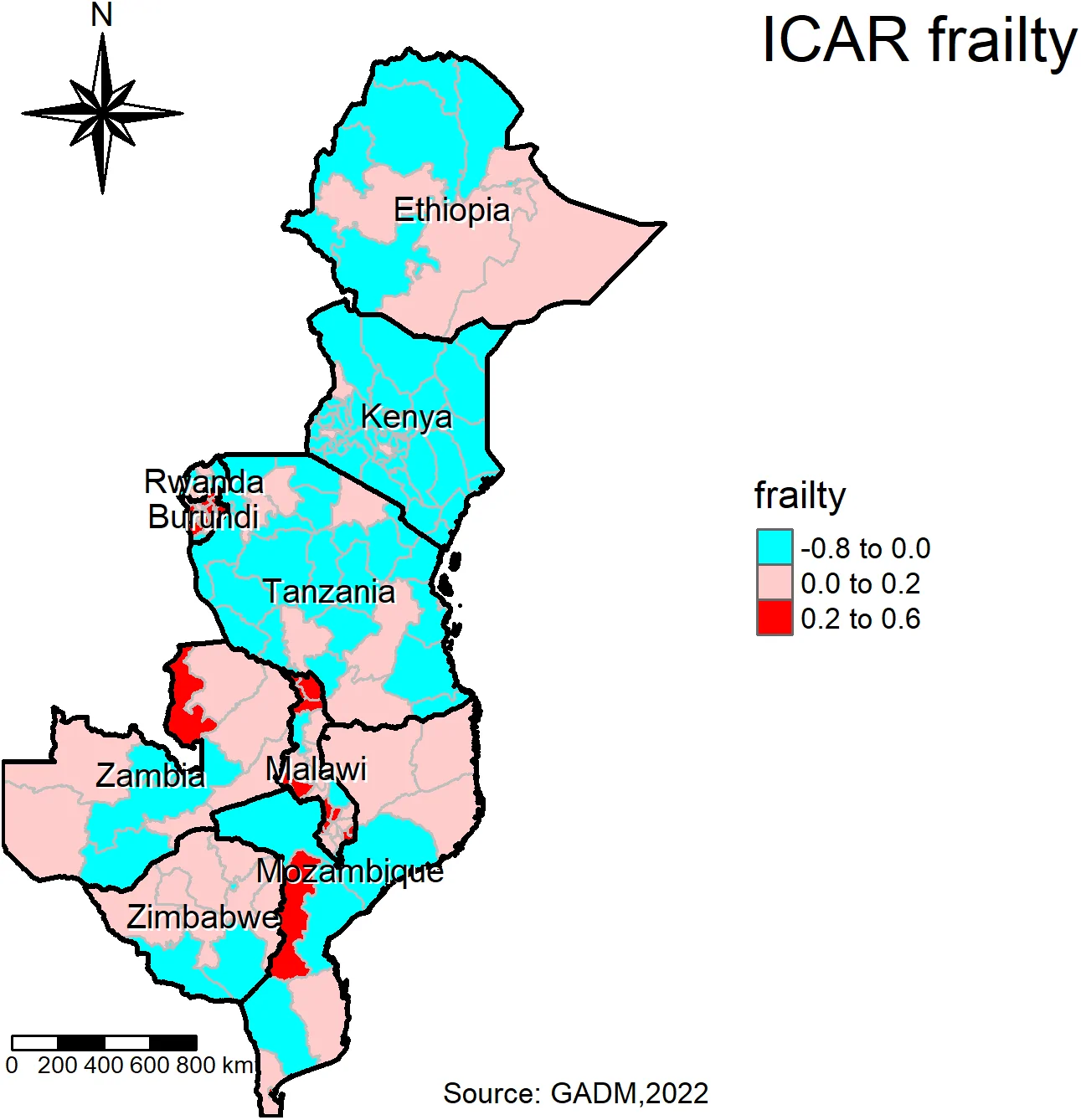
Posterior distribution of ICAR frailty for infant mortality in East Africa, DHS (2015–2022). Map created using Global Administrative Areas (GADM) data, version 4.1, freely available at (http://gadm.org). Data used under the GADM license (https://gadm.org/license.html), figure shared under CC-BY 4.0.

**Checking Convergence**

From the Bayesian analysis, the Markov Chain Monte Carlo trace plot for the coefficient, frailty, and alpha showed a stable range of values, resembling a horizontal band. There were no long upward or downward trends, and the chain frequently oscillated near the mean value of the posterior distribution. The consistent and random fluctuation of the values around the mean suggests that the MCMC had successfully converged to the posterior distribution (S2 Fig).

The Markov Chain Monte Carlo density plot showed the domination of the likelihood and the low effect of the prior in estimating the posterior distribution. The density plots for the coefficient, frailty, and alpha exhibited a smooth shape for the posterior distribution, implying that the number of iterations was sufficient for parameter estimation (S3 Fig).

## 4. Discussion

According to our study, we found the pooled estimate of the Infant Mortality Rate in East Africa to be 39.83 per 1000 live births (95% CI: 35.81, 44.27) with greater inter-country heterogeneity, as indicated by $\tau^{\text{2}}$ of 91%. This implies that 40 infants died for every 1000 live births. Our finding was consistent with evidence from East Africa [15] and the UN Inter-agency Group for Child Mortality Estimation for the least developed countries [13]. Similar socio-economic status and level of globalization might explain this [31]. However, it was higher than the UN Inter-agency Group for Child Mortality global estimate [13]. It was also higher than the study from Indonesia [32]. A high rate of infectious diseases, poor healthcare governance, low public health expenditure, and a low patient-to-physician ratio in the current study setting may be contributing factors for the disparity [31]. Additionally, study period differences might be the possible explanation. It was lower than the study conducted in Pakistan [33], Afghanistan [34], and Sub-Saharan Africa [16]. This difference could be attributed to the study periods: our study used recent data from the DHS, whereas theirs was based on older DHS data, and the ongoing conflict, insecurity, and humanitarian crises in Pakistan and Afghanistan might be the possible explanation for the disparities [34].

The spatial distribution of the ICAR frailty components revealed that there were significant differences in hazard of infant death across the study region. Specifically, northern and southwestern Malawi, western Mozambique, Zambia, and Burundi showed higher posterior frailty values. These high values imply that these areas had higher hazard of death, thus these regions were identified as the major mortality hotspots.

Even after adjusting for individual-level socio-demographic factors unmeasured contextual factor such as localized variation in healthcare infrastructure, regional poverty, and environmental stressors significantly associated with infant mortality in these areas.

The one statistically significantly associated with infant mortality was the timing of breastfeeding initiation. Our study found that infants who was breastfed after 24 hours of birth had a higher hazard of death compared to those who were breastfed immediately after birth. This finding was consistent with a systematic review and meta-analysis conducted worldwide [35], as well as a randomized controlled trial conducted in India, Tanzania, and Ghana [36]. Delaying breastfeeding initiation might result in missing out on the important nutrients and immune factors found in maternal colostrum. This might hasten death from infections, as colostrum helps protect against illnesses during infancy and later in life.

Similarly, an infant born to a mother who didn’t have at least one ANC follow-up during her pregnancy had a higher hazard of death than their counterpart. This finding was consistent with different literature, including a study conducted in India [37], a meta-analytical review of 24 developing countries [38], and a systematic review and meta-analysis conducted in Sub-Saharan Africa [39]. It might be explained by not having regular contact with a healthcare provider during pregnancy, which makes it more challenging to identify pregnancy-related complications such as gestational diabetes, preeclampsia, and infections like HIV and HBV that increase the likelihood of death [40].

The other determinant factor for infant mortality was maternal age. An infant born to a mother between the ages of 15 and 24 years had a higher hazard of death when compared with an infant born to a mother between the ages of 25 and 34. Studies conducted in Bangladesh [41], India [42], and the USA [43] report findings comparable to those observed in this study. A high incidence of infectious diseases, which is associated with early child marriage, and poor health-seeking behavior of a teenage mother might be the possible reasons for infant death [44].

According to this study, infants with low birth weight had a higher hazard of death compared to those infants with normal birth weight. This finding was consistent with the study conducted in India [45], Brazil [7], and England [46]. This could be explained by the fact that low birth weight newborns are more likely to experience birth complications such as asphyxia, improper physical growth, respiratory problems, and metabolic problems. These complications increase the risk of infectious diseases, malnutrition, and other health problems during childhood as well as in the adolescent period, which might in turn hastens death [46,47].

Plurality was the other determining factor for infant mortality. Infants born as twins or higher-order multiples had a higher hazard of death compared with singleton births. This finding aligned with a study conducted in lower and middle-income countries [48], sub-Saharan Africa [49], and Korea [50]. Poorer management of multiple pregnancies in resource-limited settings like East Africa and complications from premature birth, where multiple births are strongly associated with preterm birth, could be the possible reasons for earlier death [51]. Child sex also had a statistically significant association with infant mortality. From the findings of this study, male infants had a higher hazard of death than their female counterparts. A study from India [52] and Pakistan [53] reported similar trends with higher mortality in male infants. The male infant had a higher birth size and faced delivery complications and birth injuries; additionally, the risks of intrauterine growth restriction, prematurity, respiratory distress syndrome, and birth asphyxia are higher in male infants [54].

Parity also had a statistically significant association with infant mortality. An infant born to a mother who had more than five children had a higher hazard of death. The same finding was reported from Sub-Saharan Africa [16], a study from low- and middle-income countries [55], and Indonesia [56]. This may be due to repeated pregnancies, potentially causing damage to uterine blood vessels, which can negatively impact a child's health [56]. Additionally, higher parity correlates with increased maternal age, which can heighten the risk of pregnancy-related complications such as hypertension and gestational diabetes mellitus, further contributing to infant death [55].

Maternal employment status was significantly associated with infant mortality. An infant born to an unemployed mother had a lower hazard of death than an infant born to an employed mother. This was supported by empirical evidence from 26 developing countries. Possibly, workplace exposure to toxic elements and work-related stress would be higher for employed mothers. Additionally, exclusive breastfeeding would be better practiced among unemployed mothers [57]. While this study used a large, nationally representative dataset, which enhances the generalizability of infant mortality estimates in East Africa, it has several limitations. First, because it relied on interview-based data, important clinical variables like diseases at birth and congenital complications were not assessed. Therefore, we suggest that future research use facility-based studies to include clinical variables and contextual factors to better explain this regional variation. Additionally, because data comes from different survey years, there is some temporal variability due to changing health systems in the region. Methodologically, the potential for recall bias in retrospective birth histories might affect the precision of mortality estimates. Specifically, age heaping reporting deaths may lead to the misclassification of deaths of month and may affect the accuracy of observed trends. The Cox–Snell residual diagnostic showed departure from the 45° reference line, suggesting some remaining lack of fit in the final model. Therefore, the model estimates should be interpreted with appropriate caution.

## 5. Conclusion

Generally, from this study, the pooled infant mortality rate was 39.83 per 1000 live births (95% CI: 35.81–44.27), higher than the global estimate cutoff point. Infant mortality rate was associated with maternal, infant, and reproductive factors. In addition, high spatial frailty was observed in Malawi, the western region of Mozambique, Zambia, and Burundi, suggesting the presence of unmeasured contextual factors related to geographic location, such as disparities in health service access and living conditions. These findings highlight the policies to focus on strengthening antenatal care coverage, promoting early initiation of breastfeeding, improving maternal socioeconomic empowerment, and prioritizing high-frailty areas through resource allocation to reduce infant mortality. These findings underscore the need for region-specific interventions that address local determinants of infant mortality to reduce mortality disparities across East Africa.

## Supporting information

S1 Table Schoenfeld residual test for checking the proportional hazard assumption. (DOCX)

S1 Fig Cox-snell residual plot. (TIF)

S2 Fig MCMC trace plots for model parameter convergence. (TIF)

S3 Fig Posterior density plots for model parameters. (TIF)
