# Supplementary material for: Infant mortality rate and time to death determinants in East Africa: A Bayesian spatial frailty analysis of DHS data (2015–2022)
Source: PLoS One. 2026 Oct 5;21(10):e0357712. doi: 10.1371/journal.pone.0357712 (PMC13637874; doi:10.1371/journal.pone.0357712)
Supplement: Supplementary file 1 [file pone.0357712.s001.docx]

| **Variables** | **chi-square** | **Df** | **P value** |
| --- | --- | --- | --- |
| Sex | 7.6 | 1 | 0.006 |
| Residence | 6.35 | 1 | 0.01 |
| Maternal age | 0.65 | 2 | 0.72 |
| employment | 13.5 | 3 | 0.01 |
| plurality | 7.066 | 1 | 0.005 |
| Distance to health facility | 6.47 | 1 | 0.01 |
| Breastfeeding initiation time | 248 | 3 | 0.000 |
| Number of ANC | 0.1 | 2 | 0.99 |
| Place of birth | 7 | 1 | 0.004 |
| Parity | 0.69 | 1 | 0.40 |
| Birth weight | 2.55 | 2 | 0.46 |
| Global | 322.1 | 34 | 0.000 |
